# Antifungal activity of osthol *in vitro* and enhancement *in vivo* through Eudragit S100 nanocarriers

**DOI:** 10.1080/21505594.2017.1356503

**Published:** 2017-08-10

**Authors:** Lin-peng Li, Xiao-juan Wang, Jin-Yu Zhang, Lu-lu Zhang, Yong-bing Cao, Li-qun Gu, Yi-qun Yu, Qi-lian Yang, Chun-ying Shen, Bing Han, Yuan-ying Jiang

**Affiliations:** a Center for New Drug Research, School of Pharmacy, Second Military Medical University, Shanghai, P.R. China; b Department of Pharmacy, Minhang District Central Hospital, Shanghai, P.R. China

**Keywords:** Eudragit S100, Fluconazole-resistant *Candida albicans*, invasive candidiasis, nanoparticle, Osthol, virulence

## Abstract

*In vitro* interaction of osthol (Ost) and fluconazole (FLC) was investigated against 11 fluconazole-resistant clinical isolates of *Candida albicans*. Synergistic activities were determined using the checkerboard microdilution assay. The results of agar diffusion test confirmed the synergistic interaction. We used an enteric material Eudragit S100 for preparation of Ost nanoparticle (Ost-NP) to improve the oral bioavailability, biological activity of Ost. The physicochemical characteristics of Ost-S100-NP revealed Ost-S100-NP with mean particle size of 55.4±0.4 nm, encapsulation efficiency of 98.95±0.06%, drug loading efficiency of 23.89±0.25%, yield of 98.5±0.1% and a polydispersity index (PDI) of 0.165. As the Ost concentration-time curve showed, Ost-S100-NP can increase the plasma concentration and relative bioavailability of Ost compared with Ost-suspension by oral administration. *In vivo*, Ost-S100-NP enhanced the therapeutic efficacy of Ost against FLC-resistant *C. albicans* in immunosuppressed candidiasis mice model. The available information strongly suggests that Ost-S100-NP may be used as a promising compound against drug-resistant fungi.

## Introduction

*Candida albicans* is one of the most common fungal pathogens, mainly causing serious concern for patients with compromised immune systems such as cancer patients, transplant recipients and HIV-infected patients.^1,2^ Fluconazole (FLC) is the most widely used drug because of its high bioavailability and low toxicity.^3-6^ However, with the increasing clinical use of FLC, FLC-resistant isolates are occurring more frequently.^7,8^ To seek a novel natural product which has synergism with FLC should be encouraged.

**Figure 1.**
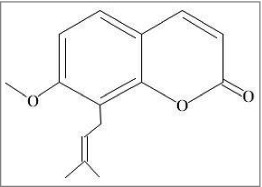
Struction of Osthol (Ost).

Osthol (Ost, Fig. 1), 7-methoxy-8-[3-methylpent-2-enyl] coumarin, is a bioactive simple coumarin derivative extracted from *Cnidium monnier* (L.) *Cusson* that has been long used in China as a herbal medicine for Gynecological infectious diseases.^9^ Recent studies have proved that Ost exhibits multiple bioactivities, including anti-allergic,^10^ neuroprotective activity,^11,12^ anticancer effect,^13-16^ cardiovascular protection,^17^ anti-inflammatory^18-21^ and antimicrobial properties.^22-25^ The clinical usefulness of Ost is limited by its poor bioavailability *in vivo*.^26-29^ According to the multiple bioactivities and metabolism of osthole, developing a new formulation of osthole as potential drug is promising.

**Figure 2.**
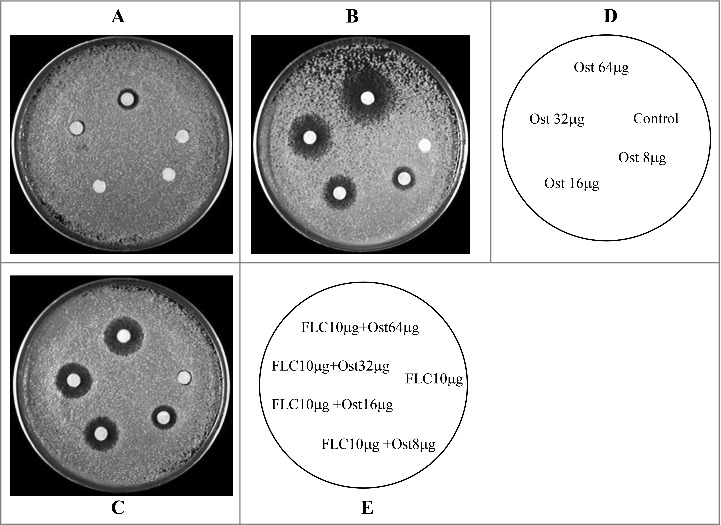
Agar disk diffusion assay of FLC combined with Ost against *C.albicans* 103. Panels A and C show plain agar plates, and panel B shows an agar plate containing 64 μg/ml of FLC. Panel D describes the images for panels A and B, and panel E describes the image for panel C.

Researches about Ost against *C. albicans* have not been reported. To seek a new combination therapy, we investigated the interaction of FLC and Ost against FLC-resistant clinical isolates of *C. albicans*. For the enhancement of low bioavailability after oral administration, we used an enteric material, Eudragit S100 for preparation of Ost nanoparticle (Ost-NP). Further, characterized the bioavailability and pharmacokinetic of Ost-NP *in vivo* and compared with Ost-suspension. *In vivo*, antifungal activity of Ost-NP was investigated by FLC-resistant *C. albicans* 103 in systematic infection mice model.

## Results

### Checkerboard microdilution assay

The results of the checkerboard analysis are summarized in Table 1. Both FLC and Ost showed weak antifungal activity when tested alone. However, the combination of FLC and Ost showed obviously inhibition of FLC-resistant *C. albicans*. Synergism was observed in all 11 isolates (100%) in terms of MIC_80_. The corresponding median FIC index was 0.135 (range, 0.047 to 0.266).

**Table 1.** Interaction of FLC and Ost against 11 clinical isolates of *C. albicans* resistant to FLC by MIC_80_s of checkerboard microdilution assay.

	MIC_80_ (μg/ml) alone		MIC_80_(μg/ml) in combination			
Clinical Isolates	FLC	Ost	FLC	Ost	FIC index for combination	Mode of Interaction
953	64	32	2	4	0.156	Syn
J35	64	32	1	8	0.266	Syn
32	64	64	2	4	0.094	Syn
904	>64	>64	2	4	0.047	Syn
557	>64	64	2	2	0.047	Syn
842	>64	64	2	2	0.047	Syn
901	>64	16	2	4	0.266	Syn
100	>64	32	4	4	0.141	Syn
103	>64	64	4	4	0.078	Syn
J28	>64	64	8	8	0.187	Syn
J5	>64	64	2	8	0.156	Syn
ATCC90028	0.25	8	0.0625	1	0.3725	Syn

### Agar diffusion test

Agar diffusion tests visualized this synergistic interaction. Ost had no anti-fungal activity in smaller dosage and showed a weak anti-fungal activity at 64 μg (Fig. 2A). FLC at 10 μg showed only weak inhibition (Fig. 2C). In contrast, Ost showed a powerful fungistatic effect on the yeast extract-peptone-dextrose (YEPD) plate containing 64 μg/ml FLC (Fig. 2B). The mean diameters of the inhibitory zones for 8, 16, 32, and 64 μg Ost increased to 8, 12, 16, and 21 mm, respectively. In addition, the combination of FLC and Ost showed significantly clearer and larger zones than FLC alone on the YEPD plate (Fig. 2C). The size of the inhibition zones increased to 9, 13, 15, and 18 mm around the disks impregnated with 10 μg FLC plus different amounts of Ost (8, 16, 32, and 64 μg), respectively.

### Physicochemical characteristics of Ost-nanoparticle

**Figure 3.**
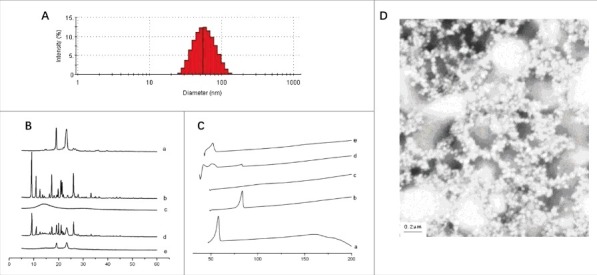
The characteristics of Ost-S100-NP. (A) Particle size distributions of Ost-S100-NP. (B) X-ray diffraction of (a) Ost (b) S100 (c) Poloxamer 188 (d) physical mixture of Ost、Eudragit S100 and Poloxamer 188 (e) Ost-S100-NP. (C) DSC spectra of (a) Ost (b) S100 (c) P188 (d) physical mixture of Ost、S100 and P188 (e) Ost-S100-NP. (D) Appearance of Ost-S100-NP under electric microscope (× 50,000).

The size distribution profiles of Ost-S100-NP was performed using a particle size analyzer with respect to intensity, volume, and number. The results revealed a wide range of Ost-S100-NP (25–120 nm in Fig. 3A) with mean size of 55.4 ± 0.4 nm, encapsulation efficiency of 98.95 ± 0.06%, drug loading efficiency of 23.89 ± 0.25%, and yield of 98.5 ± 0.1% and a polydispersity index (PDI) of 0.165. According to the PDI values, Ost-S100-NPs was moderately poly-disperse.

**Figure 4.**
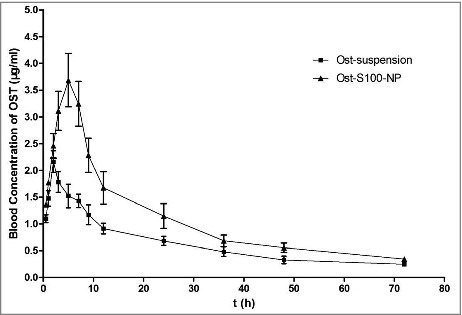
Blood concentration of profile of Ost after oral Administration of Ost-suspension(■) and Ost-S100-NP(▴) to fasted rats at a dose of 25 mg/kg (n = 8).

The presence of Ost-S100-NPs was confirmed by X-ray diffraction (XRD) (Fig. 3B) and Differential Scanning Calorimetry (DSC) (Fig. 3C). The XRD result showed that OST molecules may adhered on the particle surface. Consistent with the above results, the TEM images indicated that Ost-S100-NPs were roughly spherical and subspherical in shape, separated from each other (Fig. 3D).

### Bioavailability and pharmacokinetic studies

The mean plasma concentration-time curve of Ost by oral administration of Ost-S100-NP and Ost-suspension to rats at a single dose of 25 mg/kg are shown in Fig. 4. A 2-compartment model with a weight of 1/C^2^ assessed by AIC and correlation coefficient r of the linear equation was described the pharmacokinetics of Ost in rats, which are listed in Table 2.

**Table 2.** The Main Pharameters after Oral Administration of Ost-suspension and Ost-S100-NPs in rats. Mean ± SE (n = 8).

Parameters	Units	Ost-suspension	Ost-S100-NP
C_max_	μg·ml^−1^	1.87 ± 0.24	3.15 ± 0.29^**^
T_peak_	h	2.25 ± 0.21	4.21 ± 0.25^**^
k_a_	h^−1^	1.25 ± 0.16	0.44 ± 0.09^**^
k_10_	h^−1^	0.43 ± 0.02	0.45 ± 0.02^*^
AUC	μg·h·ml^−1^	55.47 ± 3.33	93.58 ± 4.85^**^
CL	mg·kg^−1^·h^−1^/ μg·ml^−1^	0.45 ± 0.02	0.27 ± 0.01^**^
MRT	h	22.10 ± 0.87	23.48 ± 0.64^**^
Fr(%)		—	168.7%

Notes.

* *P < 0.05* vs. Ost-suspension;

** *P < 0.01* vs. Ost-suspension.

**C_max_**: maximum concentration; **T_peak_**: time to reach C_max_; **k_a_**: the absorption constant from gastrointestinal tract to central compartment; **k_10_**: elimination constant of the central compartment; **AUC**:area under concentration curve; **CL**: clearance; **MRT**: mean retention time; Relative bioavailability: Fr % = ( AUC_Ost-S100-NP_ / AUC_Ost-suspension_) × 100%

There was a wide variability between the pharmacokinetic parameters of Ost-S100-NP and Ost-suspension. The C_max_ of Ost from Ost-S100-NP were significantly higher (3.68 ± 0.86 μg·ml^−1^) than that of Ost-suspension (2.16 ± 0.35 μg·ml^−1^), and the T_peak_(4.21 ± 0.25) was significantly longer than that of Ost-suspension (2.25 ± 0.21), while the absorption constant from gastrointestinal tract to central compartment (k_a_) decreased. The clearance (CL) of Ost-S100-NP (0.27 ± 0.01) was lower than Ost-suspension (0.45 ± 0.02), and mean residence time (MRT) of Ost-S100-NP (23.48 ± 0.64) was longer than Ost-suspension (22.10 ± 0.87). The relative bioavailability (Fr) of Ost-S100-NP was 1.69-fold higher than that from Ost-suspension. In brief, nanoparticle improved the bioavailability of Ost compared with Ost- suspension.

### *In vivo* synergism of osthole and fluconazole against candida albicans

**Figure 5.**
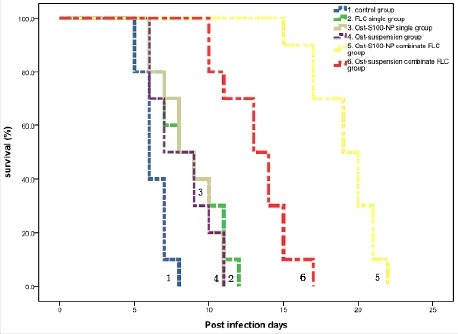
Kaplan-Meier survival curves of *C. albicans* 103 infecting mice treated with physiologic saline (1), FLC single (2), Ost-S100-NP single (3), Ost-suspensions single (4), Ost-S100-NP combinate FLC (5), and Ost-suspensions combinate FLC (6) respectively.

Figure 5 represents Kaplan-Meier survival curves. After 4 d continuous treatment, the average survival day of Ost-S100-NP plus FLC group (19.1 days) was significantly longer than saline control group (6.3 days), FLC single group (8.7 days), Ost-S100-NP single group (8.6 days), Ost-suspensions single group (8.2 days), and Ost-suspensions combinate FLC group (13.2 days). The average survival days of Ost-S100-NP single group and Ost-suspensions single group were lower than FLC single group, but there was not statistically difference while the combination groups survived significantly longer than single drug treatment groups.

## Discussion

*Cnidium monnieri* (L.) *Cusson* is a Traditional Chinese medicine which is used in Gynecological infectious diseases by traditional medicine doctors.^9^ As a major bioactivity compound of the herb, Ost has been studied extensively. Modern pharmacological studies demonstrated that it has a broad spectrum of pharmacological activities and is considered to have potential therapeutic applications such as anticancer, antifibrotic, immune-enhancing, hypotensive and antifungal activities.

Our study highlights the therapeutic potential of Ost in the treatment of FLC-resistant *C. albicans* infection. *In vitro*, checkerboard microdilution assay and agar diffusion test demonstrated that Ost can inhibit the growth of clinical isolates of *C. albicans* in combination with FLC. Ost can enhance the sensitivity of *C. albicans* to FLC.

However, the properities of Ost such as lipophilicity and poor solubility in aqueous limited the studies *in vivo*.^30^ Increasing bioavailability is necessary for the development of OST. Some approaches can be taken, including solid dispersions, microspheres,^31^ microemulsions,^32^ and nanoparticles.^33^ Nanoparticle can improve pharmacokinetics and therapeutic effects, and reduce toxicity, have received much attention as a suitable alternative system for drug delivery.^34,35^ The quasi-emulsion solvent diffusion technique is suitable to prepared nanoparticle for lipid soluble drugs. Nanoparticle carrier in this study was designed on Eudragit S100 which has been accepted as pharmaceutical excipients for oral use and are generally regards as non-toxic.^36^ After modify the parameter of temperature, stirring speed and time, concentrations of Poloxamer 188 and Eudragit S100, and ratios of alcohol/water, we prepared the best optimization of Ost-NP with a minor particle size and very high encapsulation efficiency. As the osthole concentration-time curve showed, Ost-S100-NP can increase the plasma concentration and relative bioavailability of Ost compared with Ost-suspension by oral administration.

*In vivo*, an invasive candidiasis model in immunosuppressed mice showed that Ost has synergism with fluconazole against FLC-resistant *C. albicans*. Furthermore, Ost-S100-NP enhanced the therapeutic efficacy of Ost largely. Several scholar's researches demonstrated that osthole inhibited hypha growth of *Fusarium graminearum* in glucose starvation condition,^37^ and exhibited antibacterial activities on both gram positive and gram negative bacteria.^22,24^ To date, there have been no reports of Ost in the treatment of invasive candidiasis. The rapid phase I metabolism of Ost was via the cytochrome P450 pathways, and CYP3A4 was the enzyme metabolizing Ost.^38^ But the orally administered azoles have the potential for inhibiting CYP3A4 pathways,^39^ so the combination of Ost with FLC may enhance the concentration of OST in mice. In addition, previously reported antifungal synergistic mechanisms may also be involved in the synergistic effects of osthol against FLC-resistant *C. albicans*, such as: increasing reactive oxygen species (ROS) to promote apoptosis,^40^ and inhibiting drug efflux pumps to increase intracellular drug concentration.^41,42^

In conclusion, our group showed that Ost combined with fluconazole have exerted antifungal activities on FLC-resistant *C. albicans*, this makes us optimistic about promising clinical application of this compound against drug-resistant fungi. But as a potential compound against drug-resistant fungi, the mechanisms underlying Anti-microbial properties need to be further explored.

## Materials and methods

Eleven clinical isolates of FLC-resistant *C. albicans* were used in this study, and *C. albicans* ATCC 90028 was used as a quality control. Drugs prepared in dimethyl sulfoxide (DMSO) included osthol (Shanghai Jianglai Biotechnology Co. Ltd, China) and fluconazole (Pfizer-Roerig Pharmaceuticals, New York, NY).

Eudragit S100 was supplied from Rőhm (Darmstadt, Germany). Poloxamer was supplied by the pharmaceutical plant affiliated with Shenyang Pharmaceutical University (China). High performance liquid chromatography (HPLC) grade acetonitrile, methanol, and N-hexane were purchased from China National Medicines Co. Ltd (China). Other chemicals and solvents were analytical grade.

All animals in this study were supplied from Shanghai Slac Laboratory Animal Co. Ltd (China), housed in a 12 h light/dark cycle room with appropriate temperature and humidity control. Animal experiments were done in accordance with a protocol approved by the Institutional Animal Care and Use committee (IACUC) at SMMU Health Sciences Center (SMMUHSC).

### Checkerboard microdilution assay

Assays were performed on all 11 isolates according to methods of the CLSI (formerly NCCLS) (M27-A2). The concentration of fungus suspension in RPMI 1640 medium was 10^3^ CFU/ml, and the final concentration ranged from 0.125 to 64 μg/ml for FLC and 1 to 32 μg/ml for OST. Plates were incubated at 35 °C for 24 h. Optical density was measured at 630 nm and background optical densities were subtracted from each well. MIC_80_ were defined as the minimum inhibition concentration of the drugs that inhibited growth by 80% when compared with drug-free wells. The fractional inhibitory concentration index (FICI) were defined as the sum of the MIC_80_ of each drug when used in combination divided by the MIC_80_ of the drug used alone. Synergism and antagonism were defined by FICIs of≤ 0.5 and >4, respectively. An FICI result of > 0.5 but ≤ 4 was considered indifferent.^43^

### Agar diffusion test

*C. albicans* 103 (FLC-resistant isolate with a MIC_80_ of 64 μg/ml for Ost) was tested by agar diffusion assay.^44^ A 100 μl of *C. albicans* 103 in 10^6^ CFU/ml suspension was spread uniformly onto the yeast extract-peptone-dextrose agar plate with or without 64 μg/ml FLC. Then, 6-mm paper disks impregnated with Ost and FLC alone or in combination were placed onto the agar surface. There was 5 μl of DMSO in control disks. Inhibition zones were measured after incubation at 35°C for 48 h. Assays were performed in duplicate.

### Preparation and evaluation of Ost-nanoparticle

The Ost-nanoparticle (Ost-NP) was prepared using the quasi-emulsion solvent diffusion technique (QESD). In brief, anhydrous ethanol contain Ost/Eudragit S100 (1:4, w/w) was injected rapidly into stirring water contain 125 mg Poloxamer 188 at 25°C (organic phase/aqueous phase, 2:5, v/v). The mixture was stirred at 400 rpm for 10 min, ethanol residues were left to evaporate in a 60°C water bath for 3h, resulting in the final Ost-NP preparation.

The analysis of particle size was performed by dynamic light scattering (Malvern Instruments Ltd, UK) at the wavelength of 670 nm at 25°C. Ost-NP was diluted 10-fold by ultrapure water before examination.

The concentration of Ost in the nanoparticles was determined by a reversed-phase HPLC method. The HPLC system was composed of 2 pumps (LC-10AT VP, Shimadzu, Japan) and a UV-vis detector (SPD-M10A VP, Shimadzu, Japan) set at 210 nm. The chromatographic column was a Luna C18 (5 μm in 4.6 mm × 250 mm, Phenomenex, USA) maintained at 70°C. The mobile phase consisted of acetonitrile/methanol/water (8:1:1) at a flow rate of 1.0 ml·min^−1^.

The prepared suspension of Ost-NP was filtered through a 0.45 μm filter (Shanghai Xingya cleaning materials Co., China) to remove insoluble polymer residues and Ost microcrystals. Then, 8 ml of the filtered suspension was ultracentrifuged at 250,000 × g for 60 min under 10°C and the supernatant was sampled. Ost content in the filtered suspension and in the supernatant were analyzed by HPLC.

The average particle size and size distribution (polydispersity index; PDI) was determined by Malvern Zetasizer 3000 HS. The characteristics of the NPs was observed by transmission electron microscopy (TEM) using a HITACHI EM H600 at an accelerating voltage of 110 kV. Powder X-ray diffraction (XRD) were collected on PANalytical Empyrean diffractometer operating at 30 kV, 20 mA using Cu Kα radiation with a scan speed of 0.1°/s, sampling time of 1 s, and a range of 4.8–50°. Differential Scanning Calorimetry (DSC, Diamond DSC, PerkinElmer) was used to determine the differences between Ost-S100-NP and Ost-suspensions.

The yield, encapsulation efficiency (EP%), and drug loading efficiency(LD%) were calculated using the following equations:

(1) $$Yield(\%)=\frac{c_{1}\;\times \;v}{w_{c}}\;\times \;100\%$$

(2) $$EP(\%)=\frac{(c_{1}-c_{2})\;\times \;v}{c_{1}\;\times \;v}\;\times \;100\%$$

(3) $$LD(\%)=\frac{(c_{1}-c_{2})\;\times \;v}{w_{z}}\;\;\times \;100\%$$

where, *c_1_* and *c_2_* are the drug concentrations in the filtered suspension and in the supernatant respectively, *v* is the volume of the filtered suspension *w_c_* is the theoretical amount of Ost added, and *w_z_* represents the theoretical amount of Eudragit S100 added.

### Bioavailability study

Prior to experiments, 16 male SD rats (weighing 250–300 g) were randomly separated into 2 groups (8 animals per group) and fasted overnight but with free access to water. A single oral administration dosage of Ost-suspension and Ost-S100-NP, equivalent to 25 mg/kg body weight, e. Fasting was continued for a further 4h, then at predetermined time intervals (0.5, 1, 2, 3, 5, 7, 9, 12, 24, 36, 48 and 72 h), blood samples (about 0.8 ml each) were drawn from the ocular vein into heparinized tube and centrifuged at 2600 rpm for 10 min, the plasma was removed and stored at −20°C until analyzed.

A reversed phase HPLC method was used to determine Ost concentrations in plasma. Briefly, 100 μl plasma was added to a centrifuge tube and spiked with 200 μl of internal standard (paeonolum) in acetonitrile at a concentration of 2 μg·ml^−1^. After vortexing for 3 min, it was ultracentrifuged for 6 min at 12000 × g. The supernatant was filtered through a 0.45 μm filter. 20 μl of the supernatant was injected into the HPLC system for the determination of Ost and paeonolum. The chromatographic column used was a Luna C18 (5 μm in 4.6 mm × 250 mm, Phenomenex, USA) thermostated at 25°C. The mobile phase was acetonitrile/water (60/40, v/v) with a flow rate of 1.0 ml·min^−1^.

50 μl Ost solution at concentration of 1, 2, 5, 10, 20, 30, 50 μg·ml^−1^ was added to 0.5 ml blank plasma to get the final concentrations of 0.1, 0.2, 0.5, 1.0, 2.0, 3.0, 5.0 μg·ml^−1^. These samples were also spiked with paeonolum internal standard (2 μg·ml^−1^). Quantification was done by determination of peak-area ratio of Ost/paeonolum (A/Ai) against the drug concentrations. The concentrations of unknown samples were determined using the linear regression line (unweighted) of peak-area ratios versus the concentration of the calibration standard. The regression equation of peak-area ratio of Ost/paeonolum (A/Ai): A/Ai = 15.8465C+0.0753, linear relationship was good at 0.1–5.0 ug·ml^−1^, r = 0.9993. The method recovery rate was 98.1%–104.5%. Under the described chromatographic conditions, paeonolum could be separated from Ost without any interference peaks. The retention times were 4.5 min and 6.8 min, respectively.

### Pharmacokinetic and statistical analysis

The area under concentration curve (AUC) from time zero to infinity was calculated by the trapezoidal rule method. Pharmacokinetic parameters were estimated by 3P97 (a computer program produced by the Committee of Mathematical Pharmacology of the Chinese Society of Pharmacology). These results are represented as mean ± SEM.

The statistical and graphical analyses were accomplished using commonly available commerical software packages (Microsoft Excel, Microsoft Corp., USA and GraphPad Prism). Statistical analysis was performed by t-test to compare different groups where *P<0.05* was significant.

### *In vivo* synergism of osthole and fluconazole against candida albicans

Sixty female ICR mice (weighing 20–25 g) were prepared in the antifungal study *in vivo*. We followed the methods of Junius M et al. and Kenneth et al. with a few modifications.^45,46^ Briefly, acute infections in immunosuppressed mice, resulting from a 80 mg/kg/day intravenous dose of cyclophosphamide 3 d before infection, then injected with 1 × 10^6^ CFU *C. albicans* in 0.2 ml of saline intravenously. This inoculum was uniformly lethal for placebo-treated animals within a week. After inoculation, the mice were randomly separated into 6 groups (10 animals per group), including saline control group and medication administration groups (FLC, Ost-S100-NP, Ost-suspensions, Ost-suspensions plus FLC, and Ost-S100-NP plus FLC). The dosages of FLC and Ost are 0.5 mg/kg/day and 150 mg/kg/day by oral administration. Therapy was begun 2 hours post infection and last for 4 d. The survival time was calculated from the day 0 since inoculation to the day of death. Survival data were presented as Kaplan-Merier plots and analyzed with a Log-rank Test (SPSS 18; SPSS, Chicago, Illinois). A value of *P* < 0.05 was considered to be significant.
